# Shared functional defect in IP_3_R-mediated calcium signaling in diverse monogenic autism syndromes

**DOI:** 10.1038/tp.2015.123

**Published:** 2015-09-22

**Authors:** G Schmunk, B J Boubion, I F Smith, I Parker, J J Gargus

**Affiliations:** 1 Department of Physiology and Biophysics, School of Medicine, University of California, Irvine, CA, USA; 2 Center for Autism Research and Translation, University of California, Irvine, CA, USA; 3 Department of Molecular Biology and Biochemistry, School of Biological Sciences, University of California, Irvine, CA, USA; 4 Department of Neurobiology and Behavior, School of Biological Sciences, University of California, Irvine, CA, USA; 5 Division of Human Genetics & Genomics, Department of Pediatrics, School of Medicine, University of California, Irvine, CA, USA

## Abstract

Autism spectrum disorder (ASD) affects 2% of children, and is characterized by impaired social and communication skills together with repetitive, stereotypic behavior. The pathophysiology of ASD is complex due to genetic and environmental heterogeneity, complicating the development of therapies and making diagnosis challenging. Growing genetic evidence supports a role of disrupted Ca^2+^ signaling in ASD. Here, we report that patient-derived fibroblasts from three monogenic models of ASD—fragile X and tuberous sclerosis TSC1 and TSC2 syndromes—display depressed Ca^2+^ release through inositol trisphosphate receptors (IP_3_Rs). This was apparent in Ca^2+^ signals evoked by G protein-coupled receptors and by photoreleased IP_3_ at the levels of both global and local elementary Ca^2+^ events, suggesting fundamental defects in IP_3_R channel activity in ASD. Given the ubiquitous involvement of IP_3_R-mediated Ca^2+^ signaling in neuronal excitability, synaptic plasticity, gene expression and neurodevelopment, we propose dysregulated IP_3_R signaling as a nexus where genes altered in ASD converge to exert their deleterious effect. These findings highlight potential pharmaceutical targets, and identify Ca^2+^ screening in skin fibroblasts as a promising technique for early detection of individuals susceptible to ASD.

## Introduction

Autism spectrum disorder (ASD) is a complex heterogeneous disorder^1, 2, 3, 4^ with a poorly defined etiology^5,6,7,8^ and diagnosis criteria that are strictly clinical because there are as yet no objective biomarkers of the disorder.^9, 10^ Its high heritability, however, suggests a strong genetic component,^8^ and a wealth of genetic data now implicate a host of genes encoding ion channels and associated intracellular Ca^2+^ signaling proteins in the molecular architecture of ASD,^5, 6, 7, 8^ placing Ca^2+^ homeostasis at a central node.

Cytosolic Ca^2+^ homeostasis involves ion flux from intracellular organellar stores, as well as transport across the plasma membrane. Diseases of the intracellular organelles are an emerging area of medicine. Several prototypes are already well-developed for neurogenetic diseases of mitochondria and the lysosomes,^11, 12, 13, 14^ and increasing evidence implicates the endoplasmic reticulum (ER).^15^ Ca^2+^ release from the ER through inositol trisphosphate receptors (IP_3_Rs) has been shown to be altered in cognitive disorders including Alzheimer's^16, 17^ and Huntington's diseases,^18^ and IP_3_Rs have recently been identified among the genes affected by rare *de novo* copy number variants in ASD patients.^19^

In neurons, IP_3_R-mediated Ca^2+^ release is involved in crucial functions—including synaptic plasticity and memory,^20, 21^ neuronal excitability,^22, 23^ neurotransmitter release,^24, 25^ axon growth^26^ and long-term changes in gene expression^27^—highlighting the central integrating position played by IP_3_Rs.^28^ Ca^2+^ release is activated in response to the second messenger IP_3_, which is produced on stimulation of G protein-coupled receptors (GPCRs)^29^ and tyrosine kinase-linked^30^ cell surface receptors. The specificity of the resulting cellular responses is ensured by an exquisite temporo-spatial patterning of cytosolic Ca^2+^ signals.^31, 32^ Opening of the IP_3_R channel requires not only IP_3_, but also binding of Ca^2+^ to receptor sites on the cytosolic face. This leads to biphasic regulation, such that small elevations of cytosolic Ca^2+^ induce channel opening, whereas larger elevations cause inactivation.^33^ The positive feedback by Ca^2+^ (Ca^2+^-induced Ca^2+^ release; CICR), may remain restricted to individual or clustered IP_3_Rs, producing local Ca^2+^ signals known, respectively, as Ca^2+^ blips and puffs,^34^ or may propagate throughout the cell as a saltatory wave by successive cycles of Ca^2+^ diffusion and CICR. Thus, IP_3_-mediated Ca^2+^ signaling represents a hierarchy of Ca^2+^ events of differing magnitudes.^35, 36^ The spatial patterning it orchestrates is critical to proper cellular function, and we hypothesize that disruptions in the magnitude and organization of neuronal Ca^2+^ signals may contribute to the pathogenesis of ASD.

Our understanding of the etiology of ASD ^8, 9, 37^ has been greatly advanced by studies of syndromic forms of ASD caused by rare single gene mutations. Fragile X (FXS) is the most common monogenic cause of ASD**,**^38^ and is a widely used and well-characterized model of ASD.^37, 39^ It results from silencing of the fragile X mental retardation (*FMR1)* gene and absence of its corresponding protein, the FXS mental retardation protein (FMRP). Tuberous sclerosis (TS) is a syndrome caused by dominant mutations in one of two genes, hamartin (*TSC1*) or tuberin (*TSC2*), causing ASD-like behaviors, seizures, intellectual disability and characteristic brain and skin lesions.

Here, we used primary, untransformed skin fibroblasts derived from patients with FXS and TS to evaluate ASD-associated functional deficits in IP_3_-mediated Ca^2+^ signaling. The physiology of IP_3_ signaling in fibroblasts has been extensively characterized,^40, 41, 42^ providing a validated and convenient model for the study of Ca^2+^ signaling in ASD, with the further advantage that cell lines are readily obtained as clinical samples from both disease and matched control patient populations. Moreover, identification of disease-specific signaling defects in skin cells have potential as biomarkers for diagnostic purposes, much as is now routine in other organelle diseases, such as Tay–Sachs and Niemann–Pick diseases,^43, 44^ and through which novel therapies for these diseases have emerged.^45^ Our results demonstrate that IP_3_-mediated Ca^2+^ signals are significantly depressed in fibroblasts from both FXS and TS patients and, by resolving signals at the single-channel level, we provide evidence of fundamental defects in IP_3_R channel activity in ASD. We thus propose dysregulated IP_3_R signaling as a nexus where genes altered in ASD converge to exert their deleterious effect.

## Materials and methods

### Materials

The membrane-permeant caged IP_3_ analog ci-IP_3_/PM (D-2,3-O-Isopropylidene-6-O-(2-nitro-4,5-dimethoxy)benzyl-myo-Inositol 1,4,5-trisphosphate-Hexakis (propionoxymethyl) Ester) was obtained from SiChem (Bremen, Germany), diluted in 20% pluronic F-127 solution in dimethylsulfoxide to a stock concentration of 200 μM and was frozen down into 2-μl aliquots until needed. EGTA-AM and pluronic F-127 were from Molecular Probes/Invitrogen (Carlsbad, CA, USA). Fluo-8 AM and Cal520 were purchased from AAT Bioquest (Sunnyvale, CA, USA).

### Fibroblast cells

Primary, untransformed human skin fibroblasts were purchased from Coriell Cell Repository (Camden, NJ, USA). ASD cell lines and matched controls with their corresponding Coriell numbers are as follows: FXS-1 (GM05848)/Ctr-1 (GM00498), FXS-2 (GM09497)/Ctr-2 (GM02912), FXS-3 (GM05185)/Ctr-3 (GM03440), FXS-4 (GM04026)/Ctr-4 (GM02185), FXS-5 (GM05131)/Ctr-5 (GM05659), TS1-A (GM06148)/Ctr-6 (GM01863), TS1-B (GM06149)/Ctr-3 (GM03440) and TS2 (GM06121)/Ctr-2 (GM02912). All cell lines came from male Caucasian patients. Cells were cultured in Dulbecco's Modified Eagle's Media (ATCC 30-2002; ATCC, Manassas, VA, USA) supplemented with 10% (v/v) fetal bovine serum and 1 × antibiotic mix (penicillin/streptomycin) at 37 °C in a humidified incubator gassed with 95% air and 5% CO_2_, and used for up to 15 passages. Cells were harvested in Ca^2+^, Mg^2+^-free 0.25% trypsin-EDTA (Life Technologies, Grand Island, NY, USA) and sub-cultured for 2 days before use.

### High-throughput Ca^2+^ imaging

Skin fibroblasts were seeded in clear-bottom black 96-well plates (T-3026-16; Greiner Bio One, Monroe, NC, USA) at 1.3 × 10^4^ cells per well and grown to confluency. On the day of the experiment, cells were loaded by incubation with 2 μM of the membrane-permeant Ca^2+^ indicator Fluo-8 AM^46^ in standard buffer solution (130 mM NaCl, 2 mM CaCl_2_, 5 mM KCl, 10 mM glucose, 0.45 mM KH_2_PO_4_, 0.4 mM Na_2_HPO_4_, 8 mM MgSO_4_, 4.2 mM NaHCO_3_, 20 mM HEPES and 10 μM probenecid) with 0.1% fetal bovine serum for 1 h at 37 °C, then washed with a standard buffer solution. Ca^2+^-free solution (120 mM NaCl, 4 mM KCl, 2 mM MgCl_2_, 10 mM glucose, 10 mM HEPES, 1 mM EGTA) was added to each well (100 μl), and cells were allowed to equilibrate for 5 min prior to assay with a Fluorometric Imaging Plate Reader (FLIPR; Molecular Devices, Sunnyvale, CA, USA). A basal read of fluorescence in each well (470–495 nm excitation and 515–575 nm emission, expressed in terms of a.u.) was read for 2 s. Next, 100 μl of 2 × ATP (1 μM, 10 μM, 100 μM final concentration) or 100 μl of 2 × ionomycin (to 1 μM final concentration) in Ca^2+^-free HBSS was added to each well. Only a single recording was obtained from a given well. Ionomycin-induced fluorescence changes from wells without prior addition of ATP were used to normalize ATP-evoked responses. Recordings were performed in triplicate.

### Whole-cell Ca^2+^ imaging

Cells seeded in glass-bottomed dishes were loaded for imaging using membrane-permeant esters of Fluo-8 and caged i-IP_3_ (ci-IP_3_).^47, 48^ Briefly, cells were incubated at room temperature in HEPES-buffered saline (2.5 mM CaCl_2_, 120 mM NaCl, 4 mM KCl, 2 mM MgCl_2_, 10 mM glucose, 10 mM HEPES) containing 1 μM ci-IP_3_/PM for 45 min, after which 4 μM Fluo-8 AM was added to the loading solution for further 45 min before washing three times with the saline solution. [Ca^2+^]_i_ changes were imaged using a Nikon Eclipse microscope system (Nikon, Melville, NY, USA) with a × 40 (numerical aperture=1.30) oil objective. Fluo-8 fluorescence was excited by 488-nm laser light, and emitted fluorescence (λ>510 nm) was imaged at 30 frames per s using an electron-multiplied CCD Camera iXon DU897 (Andor, Belfast, UK). A single flash of ultraviolet (UV) light (350–400 nm) from an arc lamp focused to uniformly illuminate a region slightly larger than the imaging field was used to uncage i-IP_3_, a metabolically stable isopropylidene analog of IP_3_, which evoked activity persisting for a few minutes. Image data were acquired as stack.nd2 files using Nikon Elements for offline analysis. Fluorescence signals are expressed as a ratio (Δ*F*/*F*_0_) of changes in fluorescence (Δ*F*) relative to the mean resting fluorescence at the same region before stimulation (*F*_0_). Recordings were performed in triplicate, and the measurement outcomes were compared using Mann–Whitney test.

### Imaging local Ca^2+^ events

For experiments studying local Ca^2+^ signals, cells were incubated at room temperature in HEPES buffer containing 1 μM ci-IP_3_/PM and 4 μM Cal520 for 1 h,^48^ washed and further incubated with 10 μM EGTA-AM for an another hour. Cells were then washed three times and remained in buffer for 30 min to allow for de-esterification of loaded reagents. [Ca^2+^]_i_ signals were imaged using the Nikon Eclipse microscope system described above, but now utilizing an Apo total internal reflection fluorescence × 100 (numerical aperture=1.49) oil objective. The imaging region on the camera sensor was cropped to 128x512 pixels (20.48 × 81.92 μm) to enable rapid (129 frames per s) imaging. Cal520 fluorescence (*λ*> 510 nm) was excited by 488-nm laser light within an evanescent field extending a few hundred nanometers into the cells. Image acquisition and processing was as described above for whole-cell imaging, except that local events were identified and analyzed using a custom-written algorithm based on MatLab.^48^

### Western blot analysis

Cell lines were grown in triplicates and lysed in mammalian protein extraction reagent (Thermo Scientific, Waltham, MA, USA) with complete mini protease inhibitor cocktail tablets (Roche, Dallas, TX, USA) and phosphatase 2 inhibitor cocktail (Sigma-Aldrich, St. Louis, MO, USA). Lysates were subsequently centrifuged at 14 000 r.p.m. for 15 min at +4 °C. Protein levels in the cell lysate were measured using the Bradford method.^49^ About 20 μg of protein was loaded per well with 5% β-mercaptoethanol on 3–8% gradient Tris-Acetate gels with Tris-Acetate SDS running buffer (Invitrogen) and separated by electrophoresis at 130 V. Proteins were transferred at 50 mA for 6 h to 0.2 μm nitrocellulose membranes, which were blocked in 5% nonfat milk in tris-buffered saline supplemented with 0.1% tween-20 for 1 h. Membranes were probed overnight at +4 °C with the following primary antibodies: rabbit polyclonal anti-IP_3_R1 (AB5882, Millipore, Billerica, MA, USA), rabbit polyclonal anti-IP_3_R2 (LS-C24911, LifeSpan Biosciences, Nottingham, UK), mouse monoclonal anti-IP_3_R3 (610312, BD Transduction Laboratories, Franklin Lakes, NJ, USA), rabbit polyclonal anti-IP_3_R1/2/3 (sc-28613, Santa-Cruz Biotechnology, Dallas, TX, USA), rabbit polyclonal anti-beta actin (ab8227, Abcam, Cambridge, MA, USA). Membranes were then incubated, as appropriate, with goat anti-rabbit (1:5000, Sigma-Aldrich) or goat anti-mouse (1:5000, Sigma-Aldrich) HRP-conjugated secondary antibodies for 1 h. Bands were visualized by an ImageQuant LAS 4000 imager (GE Healthcare, Uppsala, Sweden) using peroxidase substrate for enhanced chemiluminescence (ECL Prime; Amersham, Marlborough, MA, USA). Levels of protein expression were quantified via densitometry analysis using ImageJ (http://imagej.nih.gov/ij/docs/faqs.html#cite), and are expressed normalized to actin levels.

## Results

### Agonist-induced Ca^2+^ signaling is depressed in FXS and TS fibroblasts

**Figure 1:**
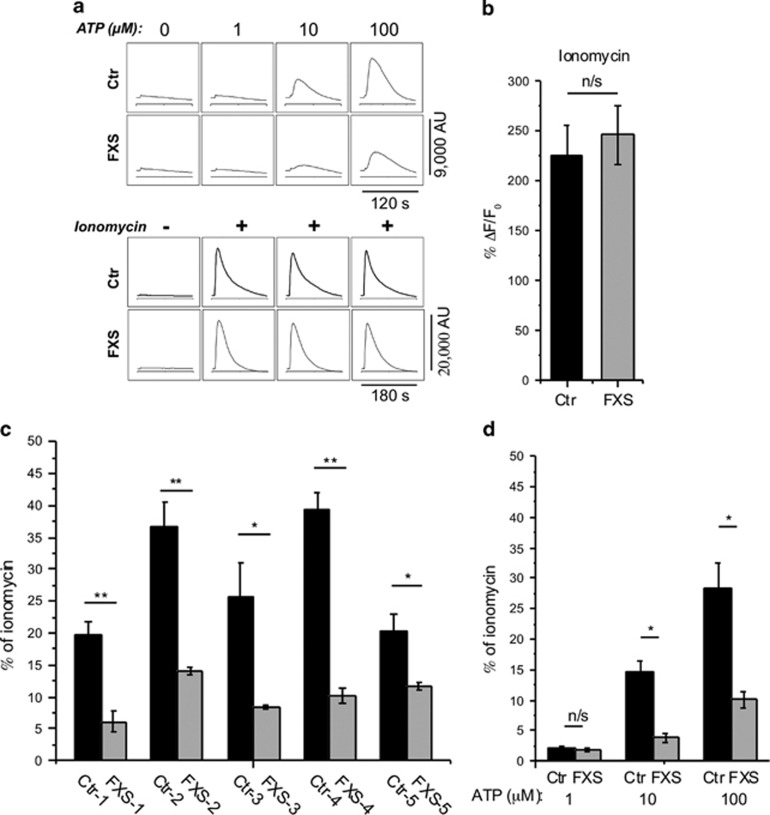
Ca^2+^ responses to extracellular application of ATP in Ca^2+^-free solution are depressed in human skin fibroblasts from FXS patients as compared with matched controls. (**a**) Representative FLIPR traces showing response to various concentrations of extracellular ATP (top panel) and to the Ca^2+^ ionophore ionomycin (lower panel) in control (Ctr) and FXS cells loaded with the Ca^2+^ indicator Fluo-8. Traces show fluorescence in arbitrary units, and each recording was obtained from a separate well. (**b**) Peak Ca^2+^ responses to 1 μM ionomycin in five independent control and five independent FXS cell lines. Bars show mean and s.e.m. of triplicate measurements on five independent cell lines; *n*=5. (**c**) Cells from five FXS cell lines (gray bars) and matched controls (black bars) were stimulated with 100 μM ATP in Ca^2+^-free solution to stimulate Ca^2+^ release from intracellular Ca^2+^ stores. Recordings were performed in triplicate, averaged and normalized with respect to corresponding ionomycin responses in Ca^2+^-free solution. *n*=3 in each group. (**d**) Normalized Ca^2+^ responses to various concentrations of ATP derived by combining results from five FXS and five matched controls. All data in this and following figures are presented as mean±s.e.m.; *P<0.05; **P<0.01 calculated from a two-sample Student's *t*-test.; FXS, Fragile X; n/s, not significant.

To screen for defects in IP_3_-mediated signaling associated with ASD, we used a FLIPR to monitor cytosolic Ca^2+^ changes in fibroblasts loaded with the Ca^2+^-sensitive fluorescent indicator Fluo-8. Primary skin fibroblasts derived from five FXS males and five ethnicity- and age-matched unaffected male donors were grown to confluency on 96-well plates. Cells were stimulated by application of ATP to activate purinergic P2Y receptors^50, 51^ and thereby evoke GPCR-mediated intracellular Ca^2+^ release through IP_3_Rs. Recordings were made in Ca^2+^-free extracellular solution to exclude complication from Ca^2+^ influx through plasmalemmal channels. Different concentrations of ATP were applied to individual wells containing FXS and matched control cells. Figure 1a (top panel) illustrates representative results, showing smaller ATP-evoked Ca^2+^ signals in FXS cells. To determine whether differences in ATP-evoked signals may result from differences in filling of ER Ca^2+^ stores, we recorded signals evoked in separate wells by application of 1 μM ionomycin in Ca^2+^-free medium to completely liberate all intracellular Ca^2+^ stores (Figure 1a, lower panel). No significant difference was observed between mean ionomycin-evoked Ca^2+^ signals in FXS and control cells (Figure 1b), suggesting that there is no systematic defect in ER Ca^2+^ store filling in FXS cells. To normalize for differences in store content among different cell lines and experimental days, we expressed ATP-evoked signals as a percentage of the ionomycin response obtained in parallel measurements in the same 96-well plate for each given cell line. Mean normalized Ca^2+^ signals evoked by 100 μM ATP were significantly depressed in all five FXS fibroblast lines in comparison to their matched controls (Figure 1c). A similar depression was observed at lower concentrations of ATP, pooling data across all five FXS and control cell lines (Figure 1d). These results were consistently reproducible across different experimental days and matched cell pairs (total of 12 paired trials).

**Figure 2:**
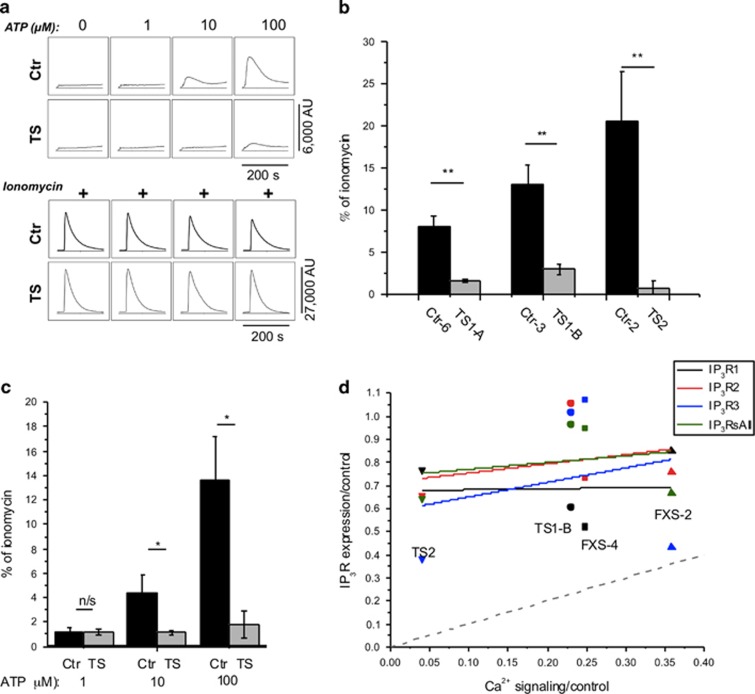
Ca^2+^ responses are strongly depressed in TS1 and TS2 fibroblasts, but IP_3_ receptor expression is not correlated with Ca^2+^ signal depression in TS or FXS cells. (**a**) Representative FLIPR traces showing response to various concentrations of extracellular ATP (top panel) and to the Ca^2+^ ionophore ionomycin (lower panel) in control (Ctr) and TS cells loaded with the Ca^2+^ indicator Fluo-8. (**b**) Three cell lines from TS patients (gray bars) and matched controls (black bars) were stimulated with 100 μM ATP in Ca^2+^-free solution to stimulate Ca^2+^ release from intracellular Ca^2+^ stores. Recordings were performed in triplicate, averaged and normalized with respect to corresponding ionomycin responses in Ca^2+^-free solution. (**c**) Normalized Ca^2+^ responses to various concentrations of ATP derived by combining results from three TS and three matched controls. *n* = 3 cell lines in each group. All data are presented as mean ± s.e.m.; *P<0.05; **P<0.01 calculated from a two-sample Student's *t*-test. (**d**) Scatter plot showing IP_3_R expression levels in TS and FXS cell lines determined by western blotting versus the mean ATP-evoked Ca^2+^ signals in these cells relative to matched control cells. Different symbols represent different cell lines (TS2, downward arrow; TS1-B, circle; FXS-2, upward arrow and FXS-4, square), and different colors represent IP_3_R expression levels as determined using antibodies for type 1 (black), type 2 (red), type 3 (blue) IP_3_Rs and a non type-specific antibody (green). All data are normalized relative to matched control cells. Solid lines are regression fits to data for IP_3_R1 (black), IP_3_R2 (red), IP_3_R3 (blue), and total IP_3_Rs (green). The gray dashed line represents a one-to-one relationship between normalized Ca^2+^ signal and normalized IP_3_R expression. FLIPR, fluorometric imaging plate reader; FXS, fragile X; IP_3_R, inositol trisphosphate receptor; n/s, not significant; TS, tuberous sclerosis.

We further extended our findings to another genetic disorder with high co-morbidity with ASD, TS, caused by mutations in either of two distinct and independent genes—hamartin (*TSC1*) or tuberin (*TSC2*). Figure 2 shows data obtained by FLIPR screening in the same way as performed for Figure 1. Three cell lines derived from TS patients demonstrated a consistent and highly significant deficit in ATP-evoked Ca^2+^ signals as compared with matched controls (Figures 2a–c), but without any appreciable difference in intracellular Ca^2+^ store content as assessed by ionomycin application (Figure 2a, lower panel). These findings were consistently replicated on different experimental days (total of six paired trials).

The diminished Ca^2+^ signals in FXS and TS cells could result from lower expression levels of IP_3_R proteins. To investigate this, we performed western blot analysis on four cell lines selected as showing pronounced defects in Ca^2+^ signaling (FXS-2, FXS-4, TS1-B and TS2), together with three matched control lines (Ctr-2, Ctr-3 and Ctr-4), using antibodies specific to type 1, 2 and 3 IP_3_Rs, as well as a non type-specific antibody (Supplementary Figure 1). Our results showed an overall slight decrease in IP_3_R expression across all isotypes in FXS and TS cells relative to their matched controls (Figure 2d). However, in all cases the depression of IP_3_R expression was much smaller than the corresponding depression of Ca^2+^ signaling as measured in the FLIPR experiments, and there was little or no correlation between IP_3_R expression and Ca^2+^ signaling in the TS and FXS cells after normalizing relative to their matched controls (Figure 2d).

### IP_3_-induced Ca^2+^ release is reduced in FXS and TS cells

To then discriminate whether the observed deficits in ATP-induced Ca^2+^ signals in FXS and TS cell lines arose through defects in any of the intermediate steps from binding to purinergic GPCR receptors to generation of IP_3_ or at the level of IP_3_-mediated Ca^2+^ liberation itself, we circumvented upstream GPCR signaling by loading cells with a caged analog of IP_3_ (ci-IP_3_).^47^ UV flash photolysis of ci-IP_3_ to photorelease physiologically active i-IP_3_ then allowed us to directly evoke Ca^2+^ liberation through IP_3_Rs in a graded manner by regulating flash duration and intensity to control the amount of i-IP_3_ that was photoreleased.

**Figure 3:**
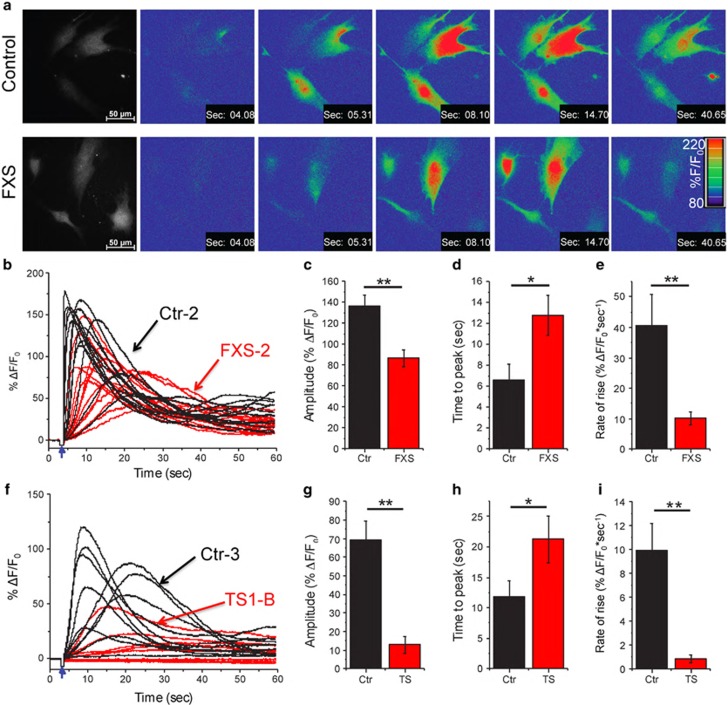
Ca^2+^ release evoked by photoreleased IP_3_ is depressed in FXS and TS cells. (**a**) Representative frames taken from image sequences of control (top) and FXS fibroblasts (bottom) loaded with Fluo-8 and stimulated by photorelease of i-IP_3_. Increasing cytosolic [Ca^2+^] (increasing fluorescence ratio %*F*/*F*_0_) is depicted on a pseudocolor scale, as indicated by the color bar. Time-stamps indicate time from beginning of the record; the photolysis flash was delivered at 3 s. The monochrome panels on the left show resting fluorescence before stimulation to indicate cell outlines. (**b**) Superimposed traces of representative global single-cell Ca^2+^ responses to uncaging of i-IP_3_ in FXS (red) and control fibroblasts (black). Traces represent average fluorescence ratio signals (%Δ*F*/*F*_0_) throughout regions-of-interest encompassing the whole cell. Arrow indicates time of the UV flash. Data are from the cell pair labeled as FXS-2/Ctr-2 in Figure 1c. (**c**) Mean peak amplitude of Ca^2+^ responses is significantly depressed in FXS cells relative to matched controls. (**d**) Mean latency from time of photolysis flash to peak IP_3_-evoked Ca^2+^ response is prolonged in FXS fibroblasts. (**e**) Mean rate of rise of Ca^2+^ fluorescence signal (peak amplitude/time to peak) is reduced in FXS cells as compared with control cells. Data in (**c–e**) are from 13 control cells and 14 FXS cells. (**f–i**) Corresponding traces (**f**), and mean values of amplitude (**g**), latency (**h**) and rate of rise (**i**) derived from cells labeled as Ctr-3 and TS1-B in Figure 2b. Data are from 11 TS cells and 12 matched controls. All data are presented as mean±s.e.m.; *P<0.05; **P<0.01 calculated from a two-sample Student's *t*-test.; FXS, Fragile X; IP_3_R, inositol trisphosphate receptor; n/s, not significant; TS, tuberous sclerosis.

Figure 3a illustrates images obtained by epifluorescence microscopy of FXS and control fibroblasts loaded with Fluo-8 and ci-IP_3_ by incubation with membrane-permeant esters of these compounds. Figure 3b shows superimposed fluorescence ratio (Δ*F*/*F*_0_) traces measured from several representative FXS-2 and matched control Ctr-2 cells in response to uniform photolysis flashes. Concordant with our observations of defects in ATP-induced global Ca^2+^ signals, global cytosolic Ca^2+^ responses evoked by equivalent photorelease of i-IP_3_ in these FXS cells were smaller than in control cells (Figure 3c); and displayed a longer time to peak (Figure 3d) and slower rate of rise (Figure 3e). Similar results were obtained from two other FXS-Ctr cell pairs (FXS-1/Ctr-1: 20.7±3.9/44.6±12.2*%ΔF/F_0_*, FXS-3/Ctr-3: 20.1±4.8/156.8±17.3). Moreover, we observed a consistent proportional depression of Ca^2+^ signals for different relative UV flash strengths corresponding to photorelease of different i-IP_3_ concentrations (25% flash strength, pooled FXS response 61% of control; 50% flash, 65% of control; 100% flash, 74% of control: *n*=13–17 cells for each flash duration).

TS cells also showed depressed and slowed Ca^2+^ responses to photoreleased i-IP_3_. Measurements from the matched TS1-B and Ctr-3 cell lines (Figure 3f) revealed a pronounced deficit in average Ca^2+^ signal amplitudes (Figure 3g); and again the time to peak was lengthened (Figure 3h) and the rate of rise slowed (Figure 3i). These differences were apparent employing two different relative UV flash strengths (15% flash strength, TS response 18% of control; 25% flash, 20% of control: *n*=13–15 cells for each flash duration).

### IP_3_-signaling is affected at the level of local events

**Figure 4:**
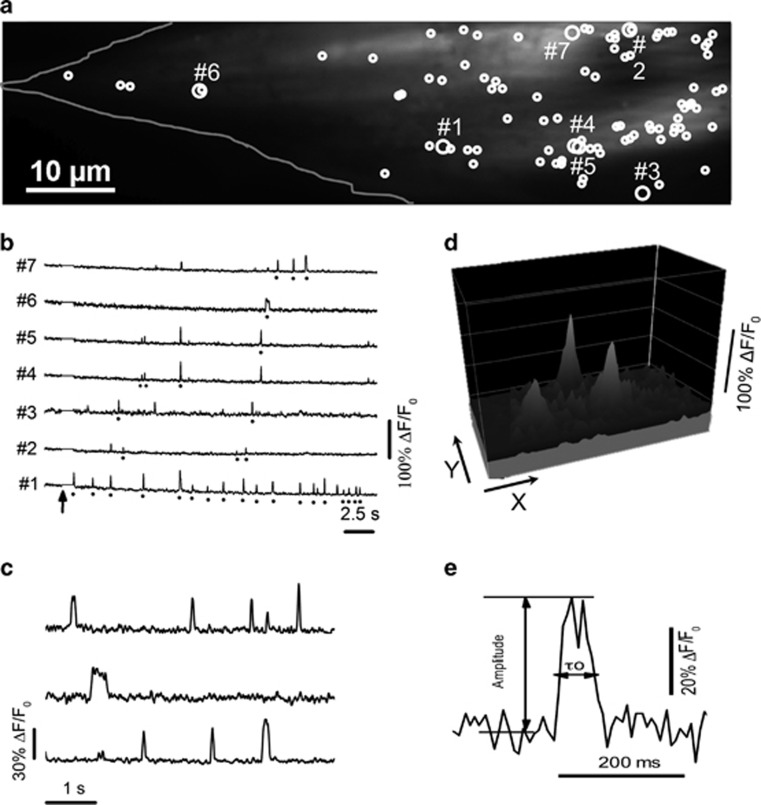
Local IP_3_-evoked Ca^2+^ events. (**a**) Resting Cal520 fluorescence of a control fibroblast (outlined) imaged by TIRF microscopy. Circles mark all sites where Ca^2+^ release events were identified within a 40 s imaging record following photorelease of i-IP_3_ in a 128 × 512 pixel (20.48 × 81.92 μm) imaging field. Larger circles mark sites from which traces in **b** were obtained. (**b**) Representative traces from sites numbered in **a**. Dots underneath the traces mark events arising at that particular site; unmarked signals represent fluorescence bleed-through from events localized to adjacent but discrete sites. Arrow indicates the timing of the UV flash. (**c**) Examples of individual events shown on an expanded timescale to better illustrate their kinetics. (**d**) Surface intensity plot of three individual puffs near their peak times. (**e**) A single Ca^2+^ event shown on an expanded scale to illustrate measurements of peak amplitude and event duration (*τ*_o_) at half-maximal amplitude. IP_3_, inositol trisphosphate; TIRF, total internal reflection fluorescence.

IP_3_-mediated cellular Ca^2+^ signaling is organized as a hierarchy, wherein global, cell-wide signals, such as those discussed above, arise by recruitment of local, ‘elementary' events involving individual IP_3_R channels or clusters of small numbers of IP_3_Rs.^34, 52^ We therefore imaged these elementary events to elucidate how deficits in the global Ca^2+^ signals in FXS and TS cells may arise at the level of local IP_3_R clusters. We selected one FXS (FXS-3) fibroblast line, one TS1 (TS1-B) line and a common control (Ctr-3) cell line matched to both. Ca^2+^ release from individual sites was resolved utilizing total internal reflection fluorescence microscopy of Cal520 (a Ca^2+^ indicator that provides brighter signals than Fluo-8), in conjunction with cytosolic loading of the slow Ca^2+^ buffer EGTA to inhibit Ca^2+^ wave propagation.^53^ This technique captures in real time the duration and magnitude of the underlying Ca^2+^ flux, providing a close approximation of the channel gating kinetics as would be recorded by electrophysiological patch-clamp recordings.^54^ Ca^2+^ release evoked by spatially uniform photolysis of ci-IP_3_ across the imaging field was apparent as localized fluorescent transients of varying amplitudes, arising at numerous discrete sites, widely distributed across the cell body (Figure 4a). Representative fluorescence traces illustrating responses at several sites (marked by large circles in Figure 4a) are shown in Figures 4b–d, respectively, and illustrate the time course and spatial distribution of selected individual events.

**Figure 5:**
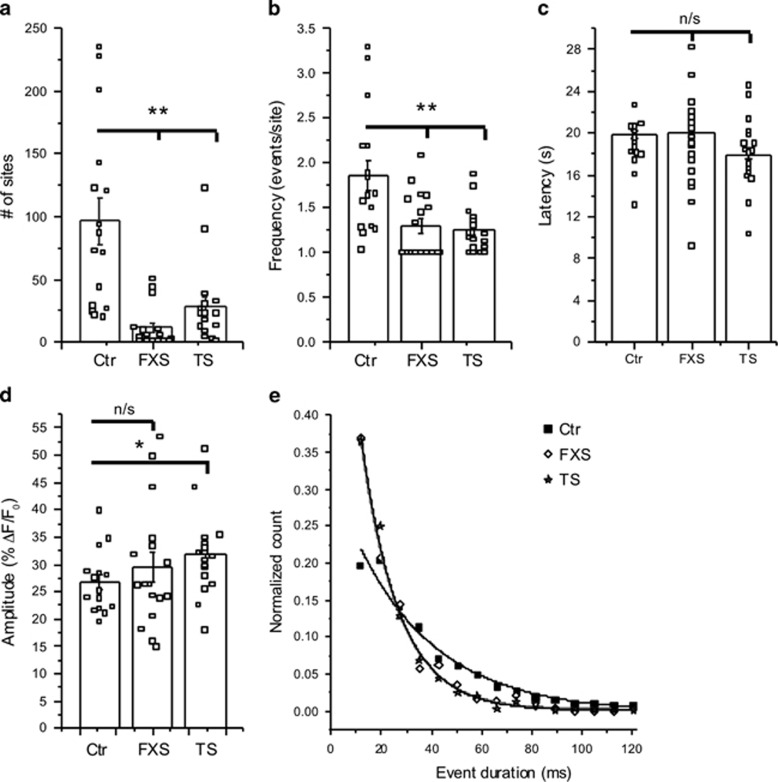
IP_3_-mediated Ca^2+^ signaling in FXS and TS fibroblasts is impaired at the level of local events. Data are from 17 FXS-3 cells, 17 TS1-B cells and 16 control cells (Ctr-3) matched to both experimental groups. Open black squares in **a**–**d** represent mean measurements from individual cells; histograms and error bars are overall mean+1 s.e.m. across all cells in each group. (**a**) Total numbers of Ca^2+^ release sites detected within cells during 40 s imaging records following uniform photorelease of i-IP_3_. (**b**) Mean event frequency per site, calculated from the number of events observed per site throughout the recording period. (**c**) Mean latencies following the photolysis flash to the first event at each site within a cell. (**d**) Mean amplitudes of all events within each cell. (**e**) Distributions of event durations (at half-maximal amplitude) derived from all events identified in FXS (open diamonds), TS (stars) and control cells (black squares). The data are fit by single-exponential distributions with time constants *t*_o_ of 15 ms (both FXS and TS) and 32 ms (control). Outcomes were compared using two-sample Mann–Whitney test. *P<0.05; **P<0.01. FXS, fragile X; IP_3_, inositol trisphosphate; n/s, not significant; TS, tuberous sclerosis.

To quantify differences in elementary Ca^2+^ events between the cell lines, we utilized a custom-written, automated algorithm^48^ to detect events and measure their amplitudes and durations (Figure 4e). A striking difference between control and ASD lines was apparent in the numbers of detected sites, with control cells showing on average 97 sites per imaging field, whereas FXS and TS cells showed only 12 and 29 sites, respectively (Figure 5a). The mean frequency of events per site appeared higher in control cells than in both FXS and TS cells (Figure 5b), but quantification was imprecise because many sites, particularly in the FXS and TS cells, showed only a single event. Using the latency between the UV flash and first event at each site as an alternative measure of the probability of event initiation^55, 56^ showed no significant difference among FXS, TS and control cell lines (Figure 5c). Mean event amplitudes were also similar among the three cell lines (Figure 5d). A second key difference between the control and FXS and TS cells was apparent in the durations of the local events. In all cell lines, event durations were statistically distributed as single-exponentials, as expected for stochastic events. However, the time constants fitted to these distributions were appreciably shorter in FXS and TS cells as compared with control cells (Figure 5e).

## Discussion

We report abnormalities of IP_3_-mediated Ca^2+^ signaling in three distinct genetic models that display high co-morbidity with ASD—FXS syndrome and two genetically-distinct forms of TS (TSC1 and TSC2). Ca^2+^ responses evoked by agonist stimulation of GPCR-mediated IP_3_ signaling were significantly smaller in fibroblasts derived from patients with FXS and TS, as compared with matched control cell lines. In contrast, we found no significant differences in Ca^2+^ liberation evoked by application of the Ca^2+^ ionophore ionomycin, indicating that the diminished responses to IP_3_ do not result from diminished ER Ca^2+^ store content. Moreover, Ca^2+^ signals evoked by intracellular uncaging of IP_3_ were depressed in FXS and TS cell lines, pointing to a deficit at the level of Ca^2+^ liberation through IP_3_Rs and not solely because of diminished GPCR-mediated production of IP_3_. Finally, we conclude that the depression of Ca^2+^ signals cannot be attributed entirely or substantially to reduced expression of IP_3_R proteins, because mean agonist-evoked Ca^2+^ responses across four FXS and TS lines were about 22% of matched controls, whereas western blots showed mean IP_3_R levels to be about 80% of controls and uncorrelated with the extent of Ca^2+^ signaling depression in these different cell lines.

By resolving Ca^2+^ liberation during ‘elementary', local signals evoked by photoreleased IP_3_,^34^ we further demonstrate that defects in global Ca^2+^ signaling in these three distinct ASD-associated models are reflected at the level of Ca^2+^ release through individual and small clusters of IP_3_Rs. In both FXS and TS cell lines, we observed fewer sites of local Ca^2+^ release as compared with a control cell line, and the durations of these events were shorter. Because functional sites are comprised of clusters of small numbers of individual IP_3_Rs, the amplitude of the fluorescence signal at a site depends on the channel permeability, together with the number of active channels in the cluster.^34^ We observed similar amplitudes of local Ca^2+^ signals across the cell lines, suggesting that the Ca^2+^-permeation properties and cluster organization of IP_3_Rs are not appreciably affected in FXS and TS. However, the shorter average duration of local events points to a modulation of IP_3_R gating kinetics, and would lead to an overall decrease in the amount of Ca^2+^ released over time. Compounding this, we found the numbers of local Ca^2+^ release sites within a cell to be dramatically lower in FXS and TS cells as compared with control cells (87% and 70%, respectively), although it is possible that the short duration events observed in the mutants may have contributed to undercounting their release sites. Taken together, our findings on local IP_3_-mediated Ca^2+^ signals indicate that the deleterious effects of FXS and TS mutations manifest at the level of the functional channel gating of IP_3_Rs, although the underlying molecular mechanism remains to be determined.

The IP_3_R is a key signaling hub in the canonical metabotropic glutamate receptor (mGluR) pathway in neurons,^20, 57^ and the mGluR theory of FXS fragile X^58^ postulates that disrupted mGluR signaling underlies the pathogenesis of the disorder. Activation of mGluRs leads to a brief hyperpolarization followed by a more prolonged depolarization.^23, 59^ The initial outward current results from the opening of small conductance Ca^2+^-activated K^+^ channels.^60, 61^ This current is proportional to the Ca^2+^ signal amplitude;^23^ and can be triggered directly by intracellular uncaging of IP_3_.^23, 59^ As a result, IP_3_-evoked Ca^2+^ release transiently hyperpolarizes the cell and briefly depresses neuronal excitability, leading to a reduction in firing frequency.^23^ Suppressed IP_3_-mediated Ca^2+^ release from the internal stores, as we report in diverse models of ASD, is thus expected to diminish the inhibitory K^+^ conductance, and as such would tend to produce neuronal hyperexcitability, consistent with observations following mGluR stimulation of ASD-model neurons.^62, 63^ A complex array of downstream signals arises from mGluR activation,^64^ whereas IP_3_R Ca^2+^ signaling is one immediate downstream target; to our knowledge its function has not yet been molecularly dissected in ASD. At present, we cannot directly extrapolate our results to IP_3_-mediated signaling in neurons, given that fibroblasts predominantly express type 3 IP_3_Rs whereas neurons predominantly express type 1 IP_3_Rs.^65^ Nevertheless, because expression levels of all three isotypes of IP_3_Rs are only slightly diminished in FXS and TS fibroblasts, we conclude that the pronounced depression of Ca^2+^ signaling does not result from diminished expression of a specific isotype. Instead, the depressed Ca^2+^ signals likely result from modulatory effects on IP_3_R function, which might extend across different isotypes.

Depression of IP_3_-mediated Ca^2+^ signaling may further disrupt neurodevelopment through separate mechanisms. IP_3_Rs have been shown to be central participants in autophagy.^66, 67, 68, 69^ Decreased levels of autophagy result in defective synaptic pruning, which has been repeatedly associated with ASD in humans and mouse models,^70^ and promotion of autophagy rescues behavioral defects in mouse models of ASD.^70^

Because of the ubiquitous nature of IP_3_R signaling and its diverse roles in almost all cells of the body, deficits in IP_3_-mediated Ca^2+^ signaling may not be limited to neurological correlates of ASD, but may also explain other characteristic ASD-associated heterogeneous symptoms, such as those of the gastrointestinal tract^71, 72^ and immune system.^73, 74^ Furthermore, since the ER serves as a sensor of a host of environmental stressors, this same mechanism may contribute to the known environmental component to the ASD phenotype, and holds the potential to reveal relevant stressors.

In conclusion, our findings indicate that ER IP_3_R signaling is affected in three distinct genetic models of ASD, pointing to the ER as a functional ‘hub' where different cellular signaling pathways merge to contribute to the pathogenesis of ASD. In addition to its role in Ca^2+^ homeostasis, the ER serves as a key integrator of environmental stressors with metabolism and gene expression, as it mediates a host of broad ranging cell stress responses such as the heat shock and unfolded protein responses.^75^ In this light it can be seen to integrate a matrix of ASD-associated risk factors. We identify the IP_3_R as a functional target in monogenic models of ASD, and we are currently exploring potential defects in IP_3_-mediated Ca^2+^ signaling in ‘typical' ASD patients without any identifiable underlying genetic cause. Ca^2+^ screening in skin fibroblasts, which are routinely acquired as clinical specimens, may thus offer a promising technique in conjunction with behavioral testing for early detection of ASD, and potentially for high-throughput screening of novel therapeutic agents.
