# Supplementary figures and images for: Shared functional defect in IP3R-mediated calcium signaling in diverse monogenic autism syndromes
Source: Transl Psychiatry. 2015 Sep 22;5(9):e643–. doi: 10.1038/tp.2015.123 (PMC5068815; doi:10.1038/tp.2015.123)

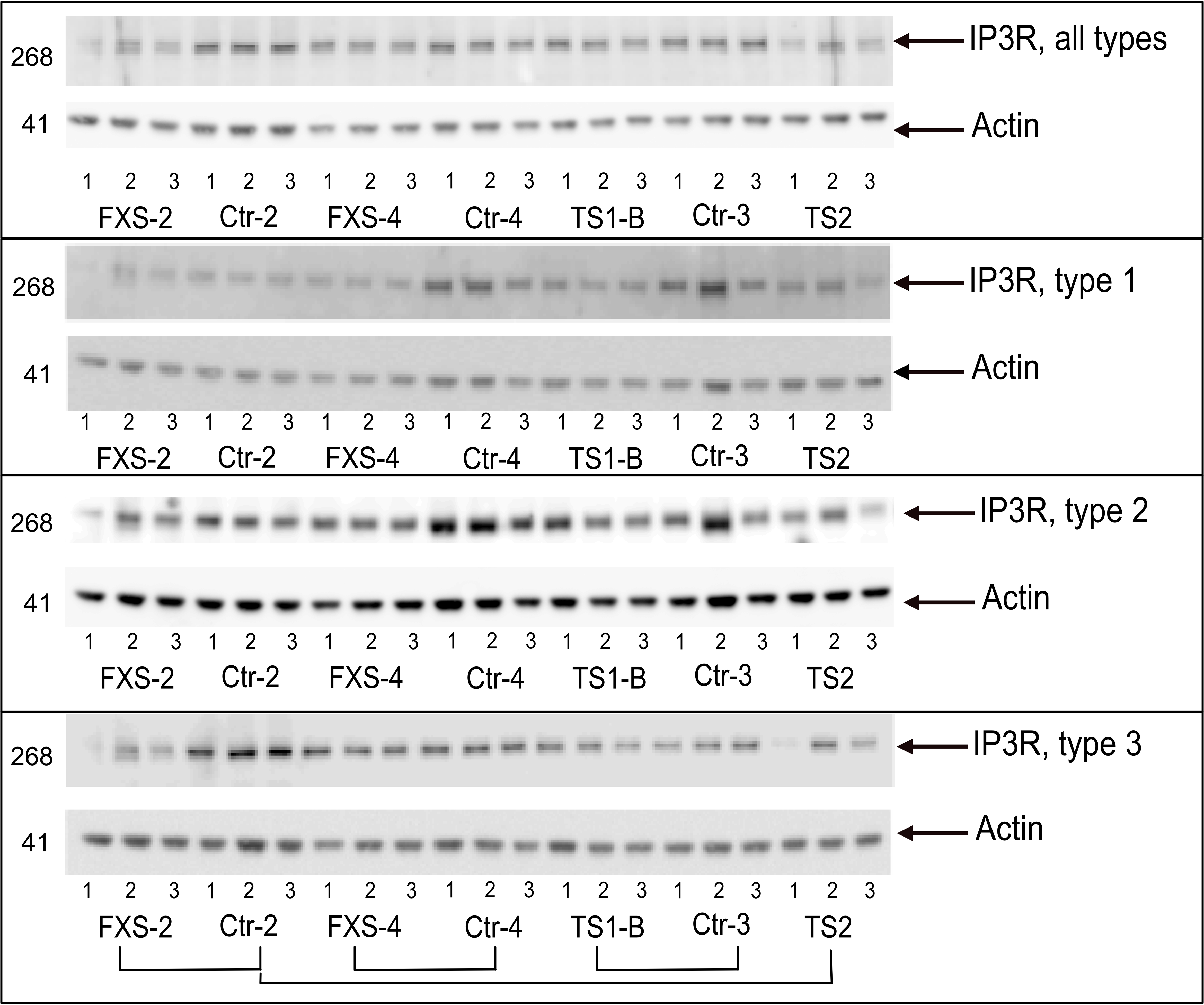

Supplement: Supplementary Figure 1 [file tp2015123x1.tif]
