# Supplementary material for: Shared functional defect in IP3R-mediated calcium signaling in diverse monogenic autism syndromes
Source: Transl Psychiatry. 2015 Sep 22;5(9):e643–. doi: 10.1038/tp.2015.123 (PMC5068815; doi:10.1038/tp.2015.123)
Supplement: Supplementary Figure Legend [file tp2015123x2.docx]

**Supplemental figure 1.** Representative immunoblots of IP_3_R proteins in skin fibroblast cell lines FXS-2, Ctr-2, FXS-4, Ctr-4, TS1-B, Ctr-3 and TS2. Aliquots of protein lysates from cell lines grown in triplicates were subjected to SDS-Tris-Acetate electrophoresis and then immunoblotted with the indicated antibodies. All IP_3_R bands ran at a molecular mass of about 270 kD. Actin was used a loading control. The leftmost lane typically showed weak transfer onto the blot, and was excluded from quantitative analysis.
